# A young adult with COVID-19 and multisystem inflammatory syndrome in children (MIS-C)-like illness: a case report

**DOI:** 10.1186/s12879-020-05439-z

**Published:** 2020-09-29

**Authors:** Aaron D. Kofman, Emma K. Sizemore, Joshua F. Detelich, Benjamin Albrecht, Anne L. Piantadosi

**Affiliations:** Emory University School of Medicine, 100 Woodruff Circle, Atlanta, GA 30322 USA

## Abstract

**Background:**

A healthy 25-year-old woman developed COVID-19 disease with clinical characteristics resembling Multisystem Inflammatory Syndrome in Children (MIS-C), a rare form of COVID-19 described primarily in children under 21 years of age.

**Case presentation:**

The patient presented with 1 week of weakness, dyspnea, and low-grade fevers, followed by mild cough, sore throat, vomiting, diarrhea, and lymph node swelling. She was otherwise healthy, with no prior medical history. Her hospital course was notable for profound acute kidney injury, leukocytosis, hypotension, and cardiac dysfunction requiring ICU admission and vasopressor support. MIS-C-like illness secondary to COVID-19 was suspected due to physical exam findings of conjunctivitis, mucositis, and shock. She improved following IVIG, aspirin, and supportive care, and was discharged on hospital day 5.

**Conclusion:**

MIS-C-like illness should be considered in adults presenting with atypical clinical findings and concern for COVID-19. Further research is needed to support the role of IVIG and aspirin in this patient population.

## Background

COVID-19 is increasingly recognized to have a protean range of clinical manifestations in adults, from respiratory illness to hyper-inflammatory and coagulopathic complications, as well as a broad spectrum of disease severity. When the epidemic began in China in late December 2019, case reports of pediatric illness were relatively rare, and almost all children had mild clinical courses. However, a growing number of reports from the United Kingdom, Italy, the United States, and elsewhere has now described a severe inflammatory syndrome in children similar to Kawasaki’s disease, a vasculitic illness of unclear etiology originally described in Japan in 1967 [1–3]. This syndrome has been named Multisystem Inflammatory Syndrome in Children (MIS-C). To date, case series of MIS-C have described multisystem organ involvement including the mucocutaneous, cardiac, gastrointestinal, and respiratory systems [4]. The mortality rate of MIS-C appears to be low, though severe illness is common, and a number of fatalities in children have been reported. Anecdotal reports of MIS-C-like illness have been reported in young adults in their early twenties, raising concern that this rare presentation of COVID-19 may also have some penetrance into younger adult age groups [5]. Herein we describe a unique case report of MIS-C-like illness in a young adult with COVID-19.

## Case presentation

A 25-year-old previously healthy woman presented to an emergency department (ED) in Atlanta, Georgia in June 2020 with a chief complaint of fatigue. She reported 1 week of weakness, dyspnea, and low-grade fevers, followed by mild cough, sore throat, vomiting, diarrhea, and lymph node swelling. She lived at home with family and had no recent travel or known sick contacts. She was a nonsmoker, drank alcohol socially, and did not use recreational drugs. She was not on any chronic medications and had no known allergies. She endorsed taking ibuprofen and acetaminophen over the prior week for symptomatic relief.

On presentation, she was afebrile, with mild hypotension (blood pressure 98/56 mmHg) and normal oxygen saturation on room air. She appeared ill, with tender cervical lymphadenopathy; significant conjunctival injection without perilimbal sparing; injected, erythematous, and cracked lips; and tenderness to palpation in the left lower abdominal quadrant. She had no rash, splenomegaly, or swelling of the extremities.

Laboratory work-up was notable for profound acute kidney injury and leukocytosis (Table 1). SARS-CoV-2 PCR from nasopharyngeal swab and SARS-CoV-2 IgG from serum were both positive. Blood cultures and legionella urine antigen was negative. The patient’s urine culture grew *Escherichia coli*, which was treated with ceftriaxone switched to piperacillin-tazobactam due to AmpC-type resistance of the isolate. Chest X-ray and CT without contrast were unremarkable. Point of care echocardiogram revealed a dilated inferior vena cava. CT abdomen/pelvis demonstrated mild peripancreatic fat stranding, felt to possibly represent acute uncomplicated pancreatitis, as well as nonspecific bilateral perinephric fat stranding. The patient was admitted to the intensive care unit (ICU) for hypotension, with diagnosis of COVID-19 and concern for possible MIS-C due to mucocutaneous, renal, GI and cardiac system involvement.

**Table 1:** Laboratory Data

	On Admission	On Discharge	Reference Range
White blood cells (10^3/mcl)	51.1	23.8	4–10
Differential count (%)			
Neutrophils	83	83	34–71
Lymphocytes	3	5	19–53
Monocytes	1	0	5–13
Eosinophils	5	2	1–7
Hemoglobin (g/dl)	11.8	8.6	13.5–17.5
Platelets (K/ul)	263	571	150–400
Sodium (mmol/L)	125	138	136–145
Potassium (mmol/L)	3.2	4.2	3.4–5.4
Chloride (mmol/L)	82	108	96–108
Carbon dioxide (mmol/L)	14	24	22–32
Blood urea nitrogen (mg/dl)	158	10	6–25
Creatinine (mg/dL)	7.74	0.55	0.5–1.2
Alanine aminotransferase (U/L)	25	20	0–40
Aspartate aminotransferase (U/L)	29	39	0–40
Lipase (U/L)	327	N/A	11–82
Troponin-I (ng/ml)	0.03	< 0.03	0–0.04
B-natriuretic peptide (pg/ml)	378	1243	0–99
Urinalysis	77 WBC/hpf, 6 squamous epithelial cells/hpf, large leukocyte esterase, negative nitrites	N/A	0–5 WBC, negative leukocyte estrate, negative nitrites
C-reactive protein (mg/L)	90	39	0–10
Erythrocyte sedimentation rate (mm/hr)	92	N/A	1–25
D-dimer (ng/ml)	960	1918	0–574
Ferritin (ng/ml)	798	N/A	11–307

The patient’s blood pressure initially normalized and her creatinine improved to 2.5 mg/dL with aggressive fluid resuscitation. She was transferred to the floor on hospital day 2, however, within 7 h she experienced recurrent hypotension requiring transfer to the ICU for the initiation of vasopressors. Workup for the new shock revealed evidence of worsening cardiac dysfunction. An electrocardiogram demonstration right axis deviation, troponin-I was newly detectable at 0.06 ng/mL and B-natriuretic peptide (BNP) increased to 1617 pg/mL. She again improved with aggressive fluid resuscitation, and quickly weaned off vasopressors while experiencing renal improvement with brisk auto-diuresis. A transthoracic echocardiogram obtained on hospital day 3 was notable for isolated right-sided ventricular dysfunction with moderate to severely reduced right ventricular systolic function, estimated right ventricular systolic pressure of 46.3 mmHg, and a flattened interventricular septum in systole consistent with right ventricular pressure overload. Left ventricular function was normal (EF 60%). CT angiogram of the chest showed mild enlargement of the main pulmonary artery up to 3.5 cm without acute pulmonary embolus, as well as interval development of scattered, peripherally localized ground-glass opacities since prior chest CT 3 days earlier. Doppler ultrasounds of all four extremities were negative for thrombi. The patient maintained normal oxygenation on room air and remained afebrile, but had persistent leukocytosis and elevated inflammatory markers.

Due to concern for inflammatory multi-system organ involvement similar to that seen in MIS-C, and risk of progression to more florid cardiac involvement, a risk-benefit discussion was held with the patient regarding treatment with intravenous immunoglobulin (IVIG), including potential risk of hypercoagulability [6]. She was treated with IVIG 2 g/kg split equally between hospital days 2 and 3 to reduce risk for thromboembolic and renal toxicities, along with aspirin 325 mg daily for 7 days, based on treatment courses suggested for pediatric patients with MIS-C or Kawasaki disease [7–9]. Additionally, the patient was offered remdesivir under an Emergency Use Authorization (EUA) basis, but declined. Her leukocytosis began to downtrend on hospital day 4, and clinical symptoms improved including conjunctivitis. She was discharged on hospital day 5 with pulmonary clinic follow-up for pulmonary hypertension, and she was treated with a 7-day course of apixaban for COVID-19-associated coagulopathy per Emory University Hospital COVID-19 treatment guidelines.

## Conclusions

The Centers for Disease Control and Prevention (CDC)‘s case definition for MIS-C is (1) an individual less than 21 years of age presenting with fever, (2) laboratory evidence of inflammation by one or more markers (such as CRP, ESR, fibrinogen), (3) evidence of clinically severe illness requiring hospitalization, with greater than 2 organ systems involved (cardiac, renal, respiratory, hematologic, GI, mucocutaneous, or neurological), (4) no other plausible alternative diagnosis, and (5) SARS-CoV-2 infection confirmed by RT-PCR, serology, or antigen testing (or, absent a positive SARS-CoV-2 test, exposure to a suspected or confirmed COVID-19 case within 4 weeks prior to symptom onset). Our patient, a previously healthy young adult woman in her mid-20’s, met these criteria with the exception of age.

Several features of our patient’s presentation raised concern for MIS-C-like illness. First, she was noted to have conjunctivitis and mucositis upon evaluation in the ED, and the cracked lips in particular were suggestive of the mucositis seen in Kawasaki’s disease. Conjunctivitis has very rarely been reported in adults with COVID-19 [10], but multiple case series of MIS-C in the pediatric population have noted this clinical feature [2, 3, 11]. Additionally, our patient had profound GI symptoms leading to hypovolemia and AKI to a creatinine of 7.7, which was fluid-responsive. While GI symptoms do occur in adults with COVID-19, they are typically less severe; by contrast, prominent GI symptoms are seen in many patients with MIS-C [4, 12]. Finally, our patient’s stable respiratory status was itself a feature shared by patients with MIS-C, who often lack intrinsic respiratory disease [2].

Other features were potentially compatible with MIS-C-like illness, including shock and cardiac dysfunction. Like many patients with MIS-C, our patient required treatment with vasopressors in the ICU; her shock was thought to be multifactorial including hypovolemic and cardiogenic. She had elevated troponin and BNP, but unlike many patients with MIS-C, her cardiac dysfunction was primarily right-sided. Echocardiogram showed severe right ventricular dysfunction and CT showed evidence of pulmonary vascular disease by enlarged PA without evidence of thrombus. It is interesting to speculate whether she had a COVID-19 related vasculitic process or diffuse microthrombi leading to elevated pulmonary vascular resistance and subsequent right ventricular strain. She did not have LV dysfunction, coronary aneurysms, or valvular dysfunction, as have been described in pediatric patients with MIS-C [2, 13].

Several other features of our patient’s clinical presentation were less consistent with MIS-C as it has been described in the pediatric population. Her profound acute kidney injury and leukocytosis were not features described in the majority of MIS-C cases described to date [4]. Additionally, her neutrophilia and lymphopenia were more consistent with typical COVID-19 findings in adults, though they have been described in cases of MIS-C as well.

In conclusion, we describe an unusual case of MISC-like illness in a young adult with COVID-19. MIS-C is an emerging and poorly understood clinical entity associated that has been described in children with COVID-19 and has features similar to Kawasaki’s disease. Children with MIS-C are increasingly treated with IVIG, aspirin, and steroids; it is not clear what if any clinical features in adults may warrant similar treatment approaches. Our patient was treated with IVIG and aspirin and improved without further cardiac involvement, but this is obviously anecdotal. Further research into COVID-19 in the young adult population is needed to better characterize the full range of clinical manifestations, and to identify potential opportunities for targeted treatment of inflammatory processes.

## Data Availability

Data sharing is not applicable to this article as no datasets were generated or analysed as a part of this case report.

## Abbreviations

COVID-19: Coronavirus disease 2019
MIS-C: Multisystem Inflammatory Disease in Children
SARS-CoV-2: Severe acute respiratory syndrome coronavirus 2
PCR: Polymerase chain reaction
RT-PCR: Reverse transcription polymerase chain reaction
IgG: Immunoglobulin G
CT: Computed tomography scan
BNP: B-type natriuretic peptide
CRP: C-reactive protein
ESR: Erythrocyte sedimentation rate
PA: Pulmonary artery
LV: Left ventricle
IVIG: Intravenous immunoglobulin
